# Preoperative evaluation of solitary pulmonary nodules and adenocarcinoma invasiveness using ultra-high-resolution computed tomography and multidimensional liquid biopsy: a prospective exploratory study

**DOI:** 10.3389/fonc.2026.1818928

**Published:** 2026-07-15

**Authors:** Zhengjun Li, Siwei Chao, Ding Li, Chang Liu, Yadong Wang, Guofeng Zhang, Sibo You, Xiaohui Zhang, Yu Wang, Yi Ren

**Affiliations:** 1 Department of Thoracic Surgery, Shenyang Chest Hospital, Shenyang, China; 2 Department of Graduate Studies, Shenyang Medical College, Shenyang, Liaoning, China; 3 Department of Operating Room, Shenyang Tenth People’s Hospital, Shenyang, Liaoning, China; 4 Detexin Biomedical (Dalian) Co., Ltd., Dalian, China

**Keywords:** early lung cancer, invasiveness prediction, multimodal diagnosis, solitary pulmonary nodule, tumor-associated autoantibodies, ultra-high-resolution CT, urinary energy metabolism

## Abstract

**Background:**

Accurate preoperative characterization of solitary pulmonary nodules (SPNs), particularly differentiation of benign from malignant lesions and assessment of adenocarcinoma invasiveness, remains challenging based on morphological features alone. We investigated whether integrating 1024-matrix ultra-high-resolution computed tomography (UHRCT) with two liquid-biopsy signals, urinary cellular energy metabolism (CEM) and a seven tumor-associated autoantibody (7-TAAb) panel, could improve diagnostic performance in surgically treated patients with SPNs.

**Methods:**

In this single-center prospective study, consecutive adults with SPNs (≤3 cm) scheduled for video-assisted thoracoscopic surgery between January 2022 and December 2024 were enrolled (n = 183). All patients underwent preoperative UHRCT (1024×1024), urinary CEM testing, and 7-TAAb testing. A paired subgroup (n = 85) additionally underwent conventional HRCT (512×512) within 1 month before surgery. Postoperative histopathology served as the reference standard. Diagnostic performance metrics were calculated for each modality, parallel-rule combined strategies, and probability-based logistic regression models.

**Results:**

Among 183 patients, 164 (89.6%) had malignant nodules. UHRCT achieved the best single-modality discrimination (AUC, 0.831; sensitivity, 87.2%; specificity, 78.9%), outperforming CEM (AUC, 0.605) and 7-TAAbs (AUC, 0.558). Under the parallel-rule strategy, multimodal testing increased sensitivity but markedly reduced specificity (sensitivity 98.8%, specificity 9.5%). The probability-based fully integrated model achieved the highest AUC (0.946). At the Youden-index threshold of 0.816, this model yielded sensitivity of 92.7%, specificity of 84.2%, PPV of 98.1%, NPV of 57.1%, and accuracy of 91.8%. In the paired comparison (n = 85), UHRCT showed higher sensitivity (92.0% vs. 68.0%) and specificity (90.0% vs. 73.3%) than HRCT. For invasiveness assessment, positivity rates of both urinary CEM (57.6% vs. 31.6%) and 7-TAAbs (53.8% vs. 31.0%) were higher in invasive adenocarcinoma than in pre-invasive lesions.

**Conclusion:**

In surgically selected patients with SPNs, 1024-matrix UHRCT provided the strongest single-modality diagnostic information, while urinary CEM and 7-TAAbs offered complementary biological signals associated with invasive adenocarcinoma pathology. Probability-based integration provided a more balanced diagnostic framework than a simple parallel-rule strategy. Given the high prevalence of malignancy and the limited number of benign lesions, these findings should be considered exploratory and require external validation in broader outpatient and screening cohorts.

## 1 Introduction

Lung cancer remains the leading cause of morbidity and mortality among all malignancies worldwide (1). With the widespread adoption of low-dose computed tomography (LDCT) screening, the detection of solitary pulmonary nodules (SPNs), particularly ground-glass nodules (GGNs), has increased significantly (2, 3). Although this has facilitated the detection of lung cancer at earlier stages, it has also introduced a critical clinical challenge: accurate preoperative stratification of nodule malignancy and invasiveness. For thoracic surgeons, accurately differentiating pre-invasive lesions (e.g., adenocarcinoma *in situ* [AIS] and minimally invasive adenocarcinoma [MIA]) from invasive adenocarcinoma (IAC) is essential for determining the optimal surgical approach and extent of resection (4, 5). However, conventional imaging features often fail to definitively differentiate these pathological subtypes, potentially leading to overtreatment of indolent lesions or inadequate resection of invasive tumors, thereby increasing the risk of recurrence.

High-resolution CT (HRCT) is currently the primary imaging modality for pulmonary nodule assessment (6). However, conventional HRCT images are typically reconstructed using a 512×512 matrix, and their spatial resolution remains constrained by physical parameters, limiting the visualization of subtle morphological features associated with microinvasion (e.g., minute solid components and intratumoral microvascular signs), resulting in a “diagnostic grey zone.” Recently, ultra-high-resolution CT (UHRCT), employing 1024×1024 or higher matrix reconstruction, has been introduced into clinical practice. By significantly reducing pixel size, UHRCT enables improved visualization of internal texture and lesion margins, providing enhanced morphological evidence for lesion characterization (7, 8). Despite these advances, CT remains inherently limited to anatomical imaging and cannot fully capture the complex biological heterogeneity of tumors (9).

To overcome the limitations of morphological imaging alone, liquid biopsy has emerged as a promising approach for assessing tumor biological activity. Tumor initiation and progression are accompanied by systemic metabolic reprogramming and immune evasion, microscopic alterations that often precede macroscopic morphological manifestations (10). Specifically, urinary cellular energy metabolites may reflect the abnormal glycolytic phenotype of cancer cells (the Warburg effect), thereby serving as noninvasive metabolic indicators. Meanwhile, the detection of a panel of seven tumor-associated autoantibodies (7-TAAbs) reflects the host’s early humoral immune response to tumor antigens, offering high specificity (11–13). Theoretically, integrating biomarkers that reflect “functional status” (metabolism and immunity) with high-precision imaging that reflects “anatomical structure” (UHRCT) could facilitate the construction of a multidimensional diagnostic model capable of overcoming the limitations of single-modality approaches.

Although each modality has shown promise in lung cancer diagnosis, prospective studies evaluating the combined value of 1024-matrix UHRCT, urinary metabolites, and serum autoantibodies for the preoperative diagnosis of pulmonary nodules remain lacking. This study aimed to investigate the diagnostic efficacy of an “imaging–immune–metabolic” multimodal joint strategy. We hypothesized that this combined model would significantly improve the discrimination between benign and malignant nodules and enable accurate assessment of invasiveness, thereby providing robust evidence to guide personalized surgical decision-making.

## 2 Methods

### 2.1 Study design and population

This prospective single-center observational study consecutively enrolled 183 patients with SPNs who underwent video-assisted thoracoscopic surgery (VATS) between January 2022 and December 2024. All enrolled patients underwent preoperative 1024-matrix UHRCT, urinary cellular energy metabolism (CEM) testing, and serum 7-TAAb testing. Postoperative histopathology served as the reference standard. A paired imaging subgroup comprising 85 patients additionally had conventional HRCT images available within 1 month before surgery, enabling within-patient comparisons between UHRCT and conventional HRCT.

The inclusion criteria were as follows: (1) age ≥ 18 years; (2) SPN ≤3 cm on chest CT; (3) planned VATS resection; and (4) availability of complete preoperative UHRCT, urinary CEM, serum 7-TAAbs, and postoperative pathological data.

The exclusion criteria were as follows: (1) multiple pulmonary nodules; (2) a history of other malignancies or preoperative anti-tumor therapy; (3) poor CT image quality precluding reliable interpretation; and (4) an indeterminate or incomplete postoperative pathological diagnosis.

**Figure 1:**
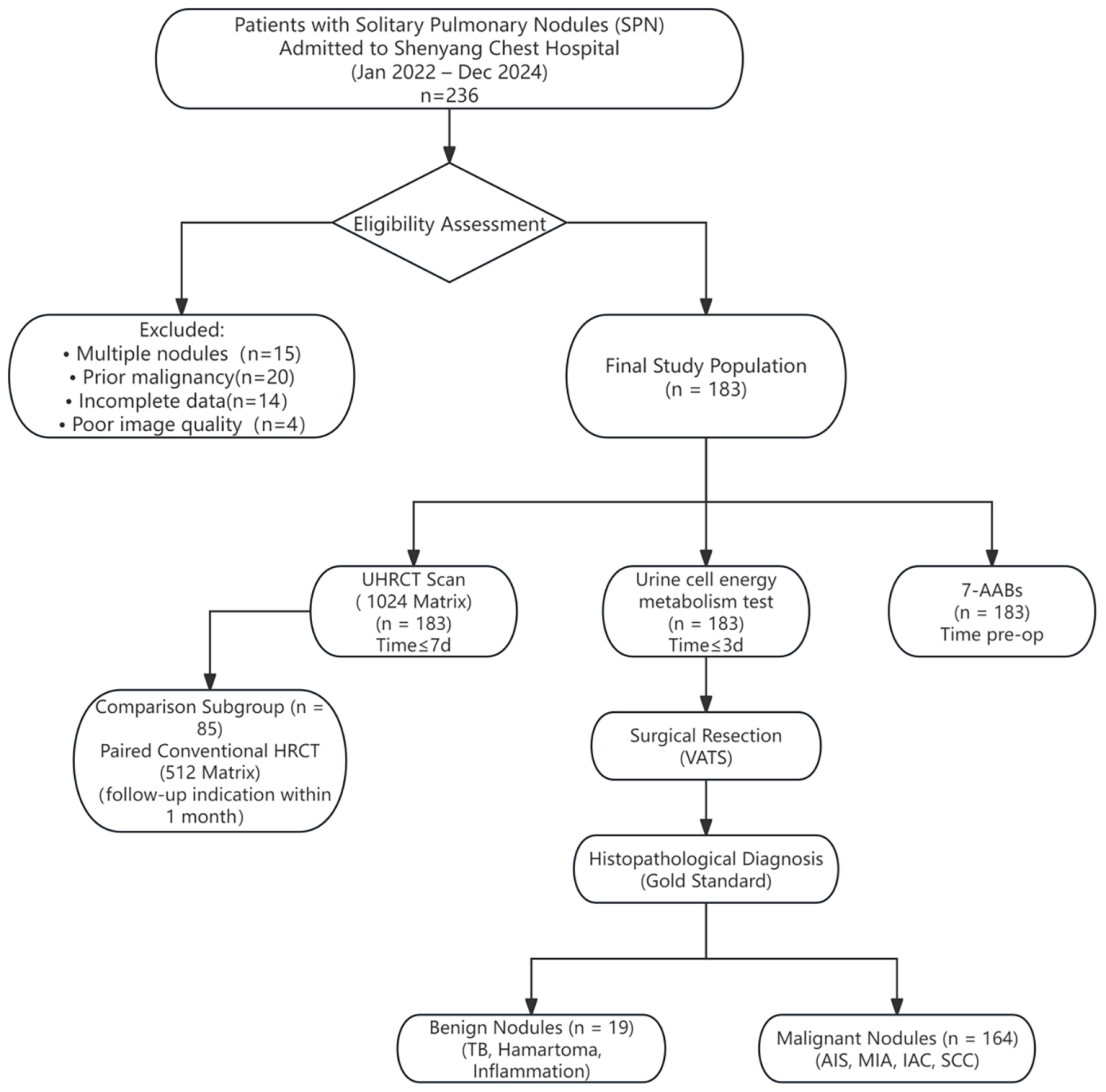
Flowchart of patient selection, examinations, and reference standard. A total of 236 patients with solitary pulmonary nodules (SPNs) admitted to Shenyang Chest Hospital between January 2022 and December 2024 were screened. After excluding patients with multiple nodules (n=15), prior malignancy (n=20), incomplete data (n=14), and poor image quality (n=4), 183 patients constituted the final study population. All included patients underwent ultra-high-resolution CT (UHRCT; 1024 matrix) within 7 days before surgery and preoperative serum seven autoantibodies (7-AABs) testing; urine cell energy metabolism testing was performed within 3 days before surgery. A comparison subgroup (n=85) additionally received paired conventional high-resolution CT (HRCT; 512 matrix) within 1 month for follow-up indication. Video-assisted thoracoscopic surgery (VATS) with histopathological diagnosis served as the reference standard, classifying nodules as benign (n=19; tuberculosis, hamartoma, inflammation) or malignant (n=164; adenocarcinoma *in situ* [AIS], minimally invasive adenocarcinoma [MIA], invasive adenocarcinoma [IAC], squamous cell carcinoma [SCC]). SPN, solitary pulmonary nodule; UHRCT, ultra-high-resolution computed tomography; HRCT, high-resolution computed tomography; 7-AABs, seven autoantibodies; VATS, video-assisted thoracoscopic surgery; TB, tuberculosis; AIS, adenocarcinoma *in situ*; MIA, minimally invasive adenocarcinoma; IAC, invasive adenocarcinoma; SCC, squamous cell carcinoma.

The participant screening process is illustrated in **Figure 1**.

### 2.2 Study objectives and evaluation strategy

Using postoperative histopathology as the reference standard, this study aimed to comprehensively evaluate the clinical value of different diagnostic modalities for early lung cancer from three perspectives:

Multimodal Diagnostic Efficacy Assessment: The diagnostic performance of urinary CEM testing, 7-TAAb testing, and UHRCT, individually and in combination, was evaluated for differentiating malignant from benign SPNs. Two types of combined analyses were prespecified: a parallel-rule combined strategy, in which the combined result was considered positive if any constituent test yielded a positive result, and probability-based logistic regression models that used predicted probabilities for receiver operating characteristic (ROC) curve and threshold analyses.

Comparative Analysis of Imaging Technologies: In the paired subgroup design, the diagnostic performance of UHRCT and conventional HRCT was compared. Sensitivity, specificity, and positive detection rates were calculated and compared to assess the advantages of UHRCT in detecting early lung cancer, particularly invasive lesions.

Biomarker Subgroup Analysis: The positive detection rates of urinary CEM and 7-TAAbs were compared between invasive adenocarcinoma (IAC) and pre-invasive adenocarcinoma-lineage lesions, including AIS and MIA.

### 2.3 Histopathological reference standard

Postoperative resection specimens were fixed and stained according to standard procedures. Histopathological diagnoses were independently established by two experienced thoracic pathologists who were blinded to the imaging and biomarker results. In cases of disagreement, a consensus was reached through consultation. Lesion classification and staging were performed in strict accordance with the 2021 World Health Organization (WHO) Classification of Thoracic Tumors and relevant guidelines from the International Association for the Study of Lung Cancer (IASLC) (14).

### 2.4 Imaging acquisition

All patients underwent chest CT within 7 d prior to surgery using a 256-slice spiral CT scanner (Brilliance iCT; Philips Healthcare, Cleveland, OH, USA). The scan range extended from the thoracic inlet to the diaphragm. Image acquisition was performed at the end of deep inspiration breath-holding according to a standard chest scanning protocol. For detailed assessment of small nodules, images were reconstructed using a 1024×1024 matrix with a slice thickness of 0.625 mm. In the paired control group, conventional HRCT images were reconstructed using a 512×512 matrix with a slice thickness of 1 mm. Raw data were stored in Digital Imaging and Communications in Medicine format for subsequent analysis.

For image interpretation for UHRCT and HRCT, two board-certified radiologists with experience in thoracic imaging independently reviewed the images. Both radiologists were blinded to the pathological results and biomarker findings (urinary metabolomics and 7-TAAbs). Each nodule was classified as benign or malignant based on an overall assessment of morphological features (e.g., lesion margins, spiculation, internal characteristics, and pleural traction), in accordance with routine clinical practice. Discrepancies were resolved through consensus review. If consensus could not be reached, a third senior thoracic radiologist served as the adjudicator. Inter-reader agreement for malignant classification was assessed using Cohen’s kappa statistic.

### 2.5 Biological sample collection and analysis

#### 2.5.1 Urinary CEM test

Patients were required to collect a morning urine sample within 3 d before surgery. All patients fasted (no food and water) for at least 8 h before sample collection. Testing was performed in strict accordance with the kit instructions (Dalian Dekesen Biomedical Co., Ltd., China).

The urinary CEM test is based on an enzyme-catalyzed reaction wherein cellular energy metabolites in urine react with specific probes to generate color. Optical density (OD) was measured at 450 nm using a microplate reader. Based on color intensity, results were semi-quantitatively classified into four grades: negative (0), weakly positive (1), positive (2), and strongly positive (3). Grades 2 and 3 were considered positive according to the manufacturer-defined cutoff used in the clinical testing protocol.

#### 2.5.2 7-TAAb test

Fasting peripheral venous blood (approximately 5 mL) was collected preoperatively from each patient. Samples were placed in pro-coagulation tubes, allowed to stand at room temperature, and centrifuged at 3000 r/min for 10 min to separate the serum. The serum was aliquoted into 1.5 mL centrifuge tubes, stored at -20 °C, and tested within 1 week.

Enzyme-linked immunosorbent assay was used to quantitatively determine the levels of seven lung cancer-associated autoantibodies: p53, PGP9.5, SOX2, GAGE7, GBU4-5, MAGE A1, and CAGE. The testing process and positive threshold determination were performed according to the manufacturer’s standard operating procedures. A sample was considered 7-TAAbs–positive if the level of at least one of the seven autoantibodies exceeded its predefined positivity threshold.

### 2.6 Statistical analysis

Statistical analyses were performed using SPSS version 22.0 (IBM Corp., Armonk, NY, USA), MedCalc version 23.4.0 (MedCalc Software Ltd., Ostend, Belgium), and R version 4.2.0 (R Foundation for Statistical Computing, Vienna, Austria). Continuous variables are presented as mean ± standard deviation or median with interquartile range, as appropriate. Categorical variables are presented as numbers and percentages.

Postoperative histopathology was used as the reference standard. For each individual diagnostic modality, including UHRCT, urinary CEM testing, and 7-TAAb testing, 2 × 2 contingency tables were constructed to calculate sensitivity, specificity, positive predictive value (PPV), negative predictive value (NPV), accuracy, and the Youden index. The 95% confidence intervals (CIs) for sensitivity, specificity, PPV, and NPV were calculated using the Wilson score method.

For the combined testing strategies, a parallel-rule approach was used.Specifically, a combined result was considered positive if any constituent test yielded a positive result. This rule represents a sensitivity-oriented strategy to reduce the likelihood of missed malignant nodules. Because such an approach may reduce specificity and increase false-positive classifications, its diagnostic performance was interpreted separately from that of the probability-based regression models.

In an additional probability-based analysis, logistic regression models were constructed to evaluate integrated diagnostic performance. Four models were evaluated: (1) a clinical model incorporating age, sex, smoking history, and nodule size; (2) a UHRCT-alone model; (3) a clinical-UHRCT model incorporating clinical variables and UHRCT; and (4) a fully integrated model incorporating clinical variables, UHRCT, urinary CEM, and 7-TAAbs. Predicted probabilities generated by these logistic regression models were used for ROC curve analysis.

The area under the ROC curve (AUC) was calculated for each probability-based model. The optimal diagnostic threshold was determined by maximizing the Youden index. Diagnostic performance at representative probability thresholds, including high-sensitivity, Youden index, and high-specificity thresholds, was further evaluated to assess the trade-off between sensitivity and specificity. Bootstrap resampling with 1, 000 repetitions was performed to assess the robustness of model performance, given the limited number of benign lesions in this surgically selected cohort.

Exploratory decision-curve analysis was performed to evaluate the potential clinical net benefit of the probability-based models across a range of threshold probabilities. Given the single-center study design and the small benign subgroup, the logistic regression and decision-curve analyses were considered exploratory.

In the paired UHRCT versus HRCT subgroup, sensitivity and specificity were compared using the McNemar test, and agreement was assessed using Cohen’s kappa statistic. In the adenocarcinoma-lineage subgroup, the positive rates of urinary CEM and 7-TAAbs were compared between invasive adenocarcinoma and AIS/MIA lesions using Fisher’s exact test. Bonferroni correction was applied when multiple pairwise comparisons were performed. All statistical tests were two-sided, and *P* < 0.05 was considered statistically significant.

### 2.7 Ethical approval of the study protocol

The studies involving human participants were reviewed and approved by Medical Ethics Committee of Shenyang Chest Hospital (KYXM-2023-015-01). Written informed consent for participation in this study was obtained from all participants or their legal guardian/next of kin. Written informed consent was also obtained from the participants and, where applicable, from the legal guardian/next of kin of minor participants for the publication of any potentially identifiable images or data included in this article.

## 3 Results

### 3.1 Baseline characteristics and pathological distribution

A total of 183 patients with SPNs who underwent surgery were included in the study. All patients underwent preoperative UHRCT, urinary CEM testing, and 7-TAAb testing. Detailed clinical and pathological characteristics are summarized in **Table 1**.

**Table 1:** Baseline clinical characteristics of the study population (N = 183).

Variable	No. ( % )
Gender	
Male	70 (38%)
Female	113 (62%)
Age (years old)	
<60	121 (66%)
≥60	62 (34%)
Smoking history	
Yes	75 (41%)
No	108 (59%)
Nodule size (cm)	
Mean ± SD	1.36 ± 0.76
Size distribution	
≤ 1.0 cm	68 (37%)
> 1.0 - 2.0 cm	73 (40%)
> 2.0 - 3.0 cm	42 (23%)
Surgical method	
Lobectomy	49 (27%)
Sublobectomy	134 (73%)
Pathological diagnosis	
Benign	19 (10%)
AIS	34 (19%)
MIA	59 (32%)
IAC	65 (36%)
SCC	6 (3%)

Data are presented as number (percentage) or mean ± standard deviation.

AIS, adenocarcinoma *in situ*; MIA, minimally invasive adenocarcinoma; IAC, invasive adenocarcinoma; SCC, squamous cell carcinoma; SD, standard deviation.

Among the 183 patients, 61.7% (113/183) were female. The mean age was 56 years, and 66.1% (121/183) were younger than 60 years of age. A history of smoking was reported in 41.0% (75/183) of patients. Regarding the surgical approach, 73.2% (134/183) of patients underwent sublobar resection.

Postoperative histopathological examination identified 164 malignant lung cancer nodules (89.6%) and 19 benign lesions (10.4%). Among malignant cases, adenocarcinoma was the predominant type. IAC was the most common subtype (35.5%, 65/183), followed by MIA (32.2%, 59/183) and AIS (18.6%, 34/183), and squamous cell carcinoma (SCC) (3.3%, 6/183).

### 3.2 Diagnostic performance of individual tests and parallel-rule combined strategies

Using postoperative histopathology as the reference standard, the diagnostic performance of the individual tests and parallel-rule combined strategies is summarized in **Table 2**. Under the parallel-rule strategy, a result was considered positive if any constituent test yielded a positive result.

**Table 2:** Diagnostic performance of single and combined models for differentiating malignant from benign SPNs.

Diagnostic model	Sensitivity (95% CI)	Specificity (95% CI)	PPV (95% CI)	NPV (95% CI)	Youden index (95%CI)
Single tests					
Urine CEM	57.9% (49.9–65.5)	63.2% (38.6–82.7)	93.1% (85.9–97.0)	14.8% (8.2–24.8)	21.1 %(11.5-48.2)
Serum 7-AABs	53.7% (45.7–61.4)	57.9% (33.9–78.9)	91.7% (83.8–96.1)	12.6% (6.8–21.9)	11.6% (0.4-40.3)
UHRCT	87.2% (80.8–91.7)	78.9% (53.9–93.0)	97.3% (92.7–99.1)	41.7% (25.9–59.1)	66.1% (34.7-84.7)
Combined models					
Urine CEM + 7-AABs	92.6% (87.1–96.0)	28.6% (12.2–52.3)	90.9% (85.2–94.6)	33.3% (14.3–58.8)	–
Urine CEM + UHRCT	95.7% (90.9–98.1)	14.3% (3.8–37.3)	89.6% (83.8–93.5)	30.0% (8.1–93.5)	–
Serum 7-AABs + UHRCT	95.0% (90.2–97.7)	19.0% (6.3–42.6)	90.0% (84.3–94.0)	33.3% (11.3–64.6)	–
Triple Combination	98.8% (95.1–99.8)	9.5% (1.7–31.8)	89.4% (83.7–93.3)	50.0% (9.2–90.8)	–

Data are presented as percentage (95% confidence interval).

CI, confidence interval; PPV, positive predictive value; NPV, negative predictive value; CEM, cellular energy metabolism; 7-AABs, seven tumor-associated autoantibodies; UHRCT, ultra-high-resolution computed tomography.

Definition of Combined Models: A combined model was defined as positive if any of the constituent tests showed a positive result (parallel testing strategy). This strategy maximizes sensitivity for early cancer detection.

Youden's Index: Youden's index was calculated as sensitivity plus specificity minus 1, ranging from -1 (perfectly wrong) to +1 (perfectly accurate), with 0 indicating a test with no diagnostic value. This metric provides a balanced measure of diagnostic accuracy by equally weighting sensitivity and specificity.

Among the individual modalities, UHRCT demonstrated the best diagnostic performance, with a sensitivity of 87.2%, specificity of 78.9%, PPV of 97.3%, NPV of 41.7%, and Youden index of 66.1%. In contrast, urinary CEM testing yielded a sensitivity of 57.9% and specificity of 63.2%, whereas 7-TAAb testing showed a sensitivity of 53.7% and specificity of 57.9%.

Under the parallel-rule combined strategy, sensitivity increased with multimodal combination, but specificity decreased substantially. The combinations of urinary CEM + UHRCT and 7-TAAbs + UHRCT yielded sensitivities of 95.7% and 95.0%, respectively; however, their specificities decreased to 14.3% and 19.0%, respectively. The triple-modality parallel-rule strategy achieved the highest sensitivity (98.8%), but its specificity was only 9.5%. These results indicate that the parallel-rule approach was highly sensitivity-oriented but was associated with a marked increase in false-positive classifications.

### 3.3 Probability-based integrated logistic regression model and threshold analysis

**Figure 2:**
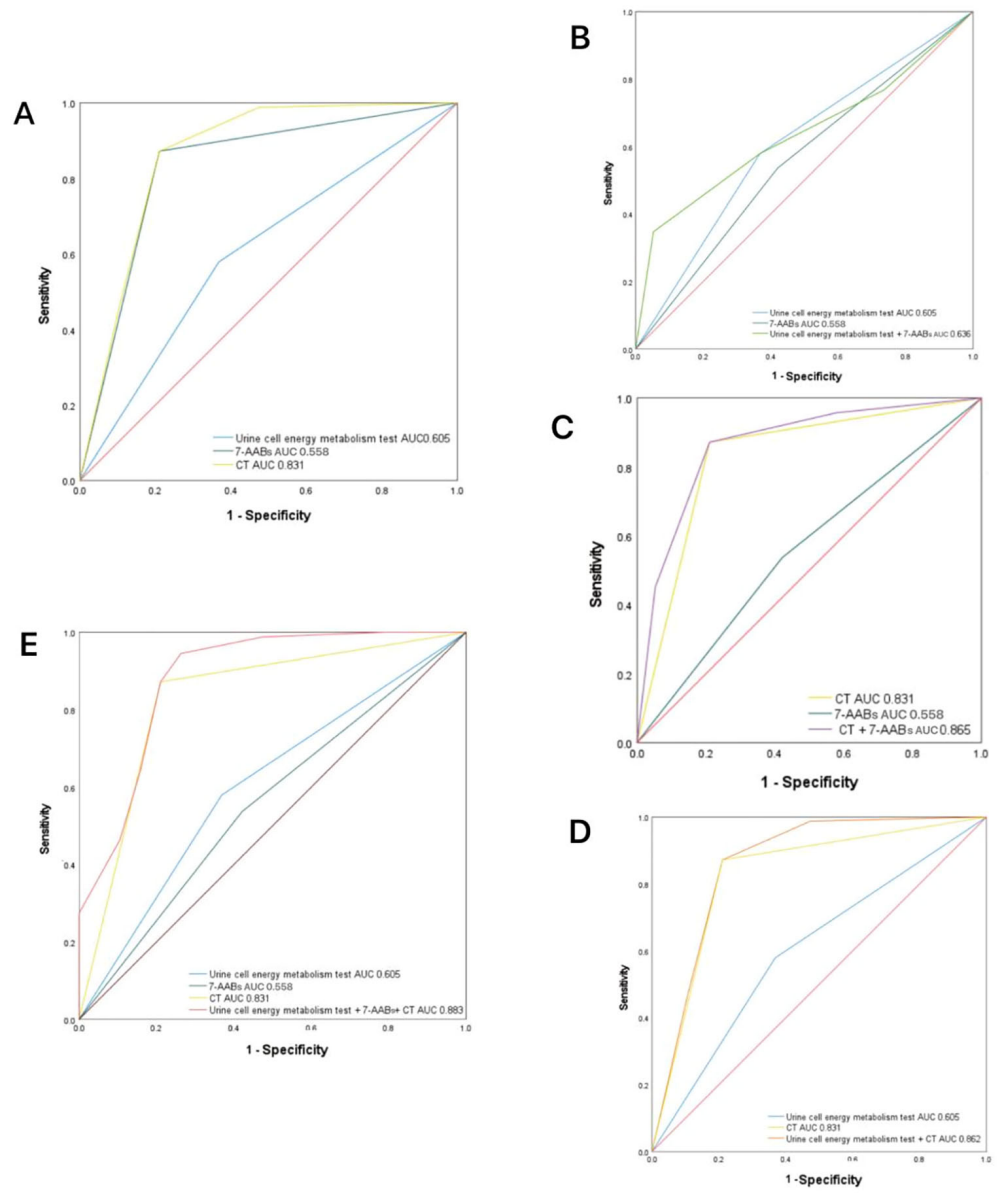
Receiver operating characteristic (ROC) curves illustrating the diagnostic performance of single and multimodal models for differentiating malignant from benign solitary pulmonary nodules. **(A)** Single diagnostic modalities: UHRCT (yellow line, AUC = 0.831) demonstrated superior performance compared to the Urine cellular energy metabolism test (blue line, AUC = 0.605) and 7-AABs (green line, AUC = 0.558). **(B)** Liquid biopsy combination: The combination of Urine and 7-AABs (green line) yielded an AUC of 0.636. **(C)** Immune-imaging dual model: Adding 7-AABs to UHRCT (purple line) improved the AUC to 0.865. **(D)** Metabolic-imaging dual model: Adding the Urine test to UHRCT (orange line) improved the AUC to 0.862. **(E)** Triple-combination model: The integrated model combining UHRCT + Urine + 7-AABs (red line) achieved the highest diagnostic efficacy with an AUC of 0.883, outperforming single modalities. ROC, receiver operating characteristic; AUC, area under the curve; UHRCT, ultra-high-resolution computed tomography; 7-AABs, seven tumor-associated autoantibodies.

**Figure 3:**
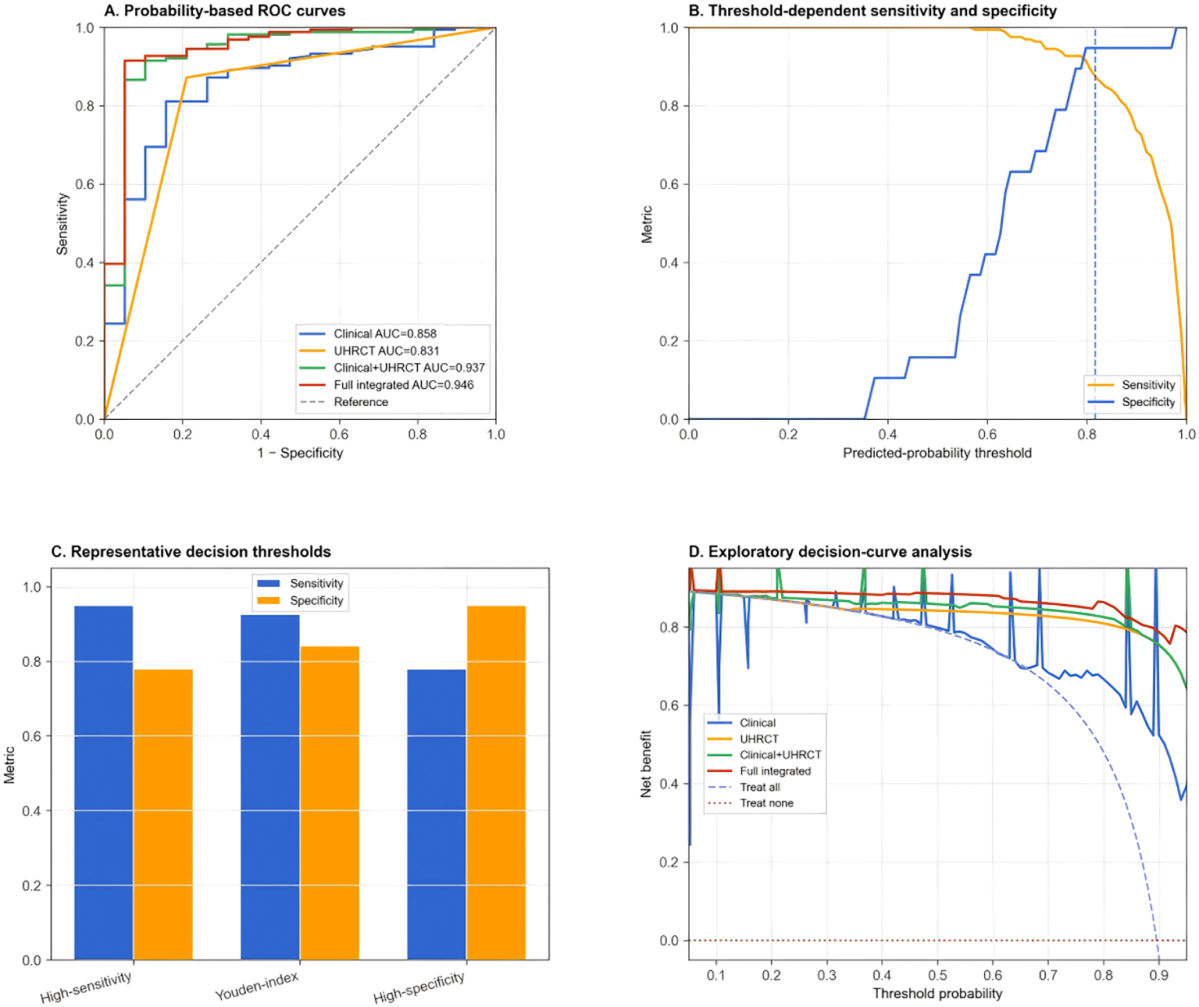
Probability-based integrated logistic regression model and threshold-dependent diagnostic performance for malignant SPN discrimination. **(A)** Receiver operating characteristic (ROC) curves for probability-based logistic regression models, including a clinical model, UHRCT alone, a clinical-UHRCT model, and the full integrated model. The full integrated model incorporated age, sex, smoking history, nodule size, UHRCT, urinary cellular energy metabolism (CEM), and 7-TAAbs. Note: These ROC curves are derived from predicted probabilities of logistic regression models and are distinct from the parallel-rule combination analysis presented in **Figure 2**. **(B)** Threshold-dependent changes in sensitivity and specificity across different predicted-probability cutoffs in the full integrated model, illustrating the trade-off between false-negative and false-positive classifications. **(C)** Diagnostic performance of the full integrated model at representative decision thresholds, including high-sensitivity, Youden-index, and high-specificity thresholds. At the Youden-index threshold of 0.816, the model achieved a sensitivity of 92.7% and a specificity of 84.2%. **(D)** Exploratory decision-curve analysis evaluating the potential clinical net benefit of the probability-based models across a range of threshold probabilities. SPN, solitary pulmonary nodule; ROC, receiver operating characteristic; AUC, area under the curve; UHRCT, ultra-high-resolution computed tomography; CEM, cellular energy metabolism; 7-TAAbs, seven tumor-associated autoantibodies.

To address the limited specificity of the parallel-rule combined strategy, we further constructed probability-based logistic regression models incorporating clinical variables and diagnostic modalities. Unlike the parallel-rule strategy described in Section 3.2, these models incorporated clinical variables (age, sex, smoking history, and nodule size) and generated continuous predicted probabilities through logistic regression, which explains the higher AUC values observed in this section compared with the parallel-rule combination results in **Figure 2**. The clinical model, which included age, sex, smoking history, and nodule size, achieved an AUC of 0.858. UHRCT alone yielded an AUC of 0.831(**Table 3**, **Figure 3A**). When clinical variables were combined with UHRCT, the AUC increased to 0.937. The fully integrated model, incorporating clinical variables, UHRCT, urinary CEM, and 7-TAAbs, achieved the highest AUC of 0.946. The results of the multivariable logistic regression analysis, including odds ratios and 95% CIs for each predictor, are presented in **Supplementary Table 1**.

**Table 3:** Diagnostic performance of probability-based logistic regression models for differentiating malignant from benign solitary pulmonary nodules.

Model	Variables included	AUC	Youden index	(95%CI)	Sensitivity	Specificity	PPV	NPV
Clinical model	Age, sex, smoking history, nodule size	0.858	85.6%	82.3%	84.2%	97.8%	35.6%	82.5%
UHRCT alone	UHRCT result	0.831	97.3%	87.2%	78.9%	97.3%	41.7%	86.3%
Clinical + UHRCT	Clinical variables + UHRCT	0.937	92.8%	81.7%	94.7%	99.3%	37.5%	83.1%
Full integrated model	Clinical variables + UHRCT + urinary CEM + 7-TAAbs	0.946	81.6%	92.7%	84.2%	98.1%	57.1%	91.8%

Clinical variables included age, sex, smoking history, and nodule size. Predicted probabilities were generated using logistic regression. The optimal threshold was determined by maximizing the Youden index. PPV and NPV should be interpreted cautiously because of the high malignancy prevalence in this surgically selected cohort. UHRCT, ultra-high-resolution computed tomography; CEM, cellular energy metabolism; 7-TAAbs, seven tumor-associated autoantibodies; AUC, area under the curve; PPV, positive predictive value; NPV, negative predictive value.

At the Youden-index threshold of 0.816, the fully integrated model yielded a sensitivity of 92.7%, specificity of 84.2%, PPV of 98.1%, NPV of 57.1%, and overall accuracy of 91.8%. Threshold-dependent changes in sensitivity and specificity are shown in **Figure 3B**. Representative threshold-specific diagnostic performances, including high-sensitivity, Youden-index, and high-specificity thresholds, are presented in **Figure 3C**. An exploratory decision-curve analysis of the probability-based models is shown in **Figure 3D**.

These findings indicate that probability-based thresholding provided a more balanced diagnostic performance than the sensitivity-oriented parallel-testing rule, particularly by improving specificity while maintaining high sensitivity.

### 3.4 Diagnostic performance comparison between UHRCT and HRCT

**Figure 4:**
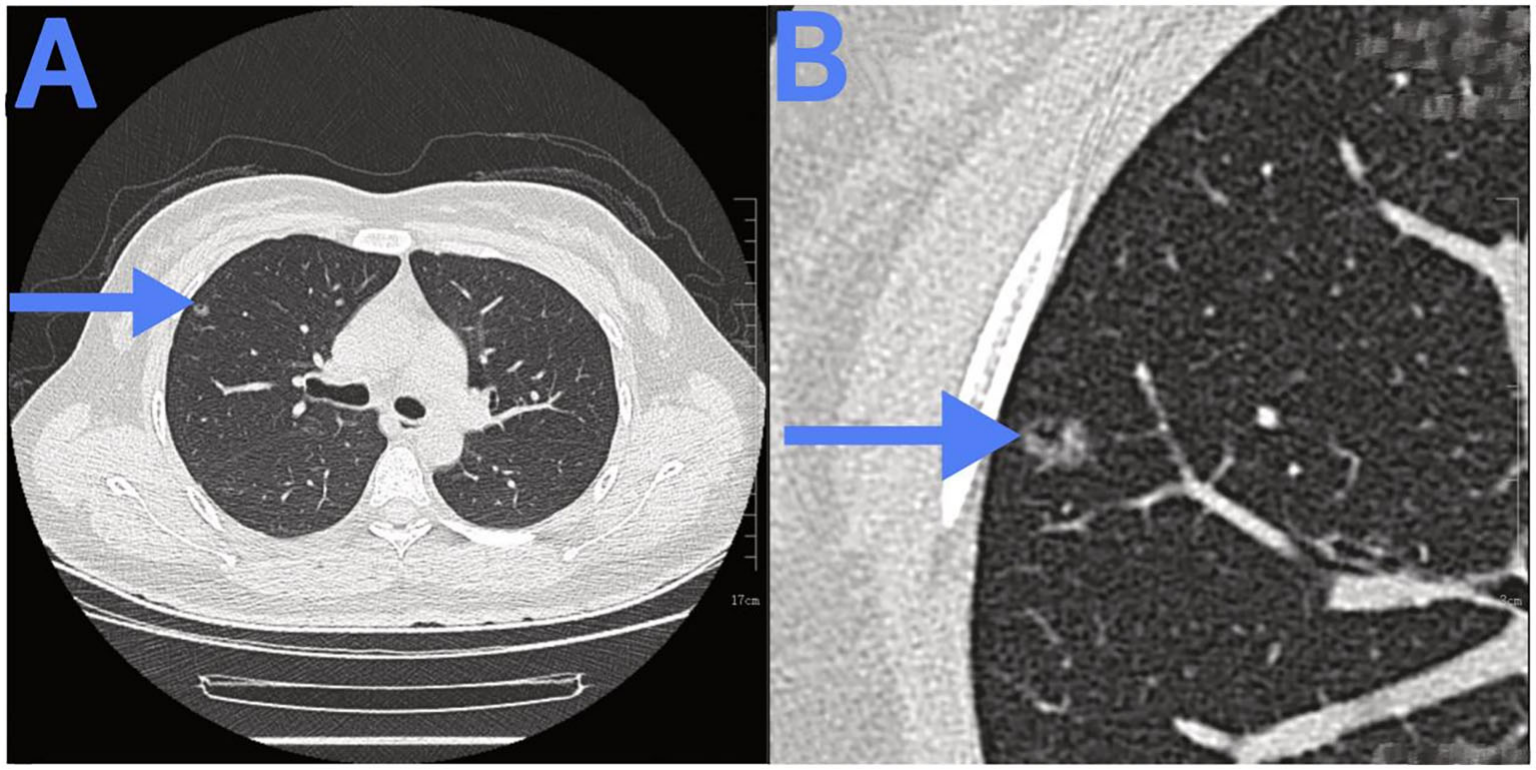
Representative comparison of image quality and nodule visualization between conventional HRCT and ultra-high-resolution CT (UHRCT) in the same patient. **(A)** Conventional HRCT image (512 × 512 matrix) showing a ground-glass nodule (GGN) in the right lung (blue arrow). Note that the internal structure and marginal characteristics appear relatively blurred due to the standard resolution. **(B)** UHRCT image (1024 × 1024 matrix) of the same lesion with a targeted field of view. The increased spatial resolution significantly enhances the visualization of morphological details. The blue arrow points to the nodule, revealing sharper margins and clearer internal features compared to **(A)**. Pathological diagnosis: The postoperative pathology confirmed this nodule as Minimally Invasive Adenocarcinoma (MIA). HRCT, high-resolution computed tomography; UHRCT, ultra-high-resolution computed tomography.

In the paired subgroup of 85 patients who underwent both UHRCT and HRCT, UHRCT demonstrated significantly superior diagnostic performance compared with conventional HRCT (**Table 4**; **Figure 4**). UHRCT achieved a sensitivity of 92.0% (95% CI: 72.5%-98.6%) and specificity of 90.0% (95% CI: 78.9%-95.9%), both outperforming HRCT’s sensitivity of 68.0% (95% CI: 46.4%-84.3%) and specificity of 73.3% (95% CI: 60.1%-83.5%). Statistical analysis showed that the positive detection rate of UHRCT (90.6%) was significantly higher than that of HRCT (71.8%) (*P*’ < 0.025). These findings highlight the advantage of UHRCT in the differential diagnosis of early lung cancer and suggest its potential to improve follow-up detection and reduce missed diagnoses.

**Table 4:** Comparison of diagnostic performance between UHRCT and conventional HRCT in the paired subgroup (n=85).

Imaging Modality	Sensitivity (95% CI)	Specificity (95% CI)	PPV (95% CI)	NPV (95% CI)
UHRCT (1024 matrix)	92.0% (72.5–98.6)	90.0% (78.9–95.9)	79.3% (59.7–91.3)	96.4% (86.6–99.4)
HRCT (512 matrix)	68.0% (46.4–84.3)	73.3% (60.1–83.5)	51.5% (33.9–68.8)	84.6% (71.4–92.7)
P-value	P< 0.05	P< 0.05	–	–
P-value'	P< 0.025	P< 0.025	–	–

Data are presented as percentage (95% confidence interval). The comparison was performed on a subset of 85 patients who underwent both scans.

UHRCT, ultra-high-resolution computed tomography; HRCT, high-resolution computed tomography; CI, confidence interval; PPV, positive predictive value; NPV, negative predictive value.

### 3.5 Biomarker subgroup analysis

Among the 164 malignant nodules, 158 were lung adenocarcinoma–lineage lesions (AIS/MIA/IAC) and 6 were SCC. Analysis of tumor invasiveness was therefore restricted to adenocarcinoma-lineage lesions. Using postoperative histopathology as the reference standard, the positive detection rates of urinary CEM and 7-TAAbs between IAC and pre-invasive lesions (AIS/MIA) were compared. The results showed that in the IAC group, the positive rates for urinary CEM and 7-TAAbs were 57.6% and 53.8%, respectively. In the pre-invasive group, the corresponding rates were 31.6% and 31.0%, respectively. Positive detection rates for both biomarkers were significantly higher in the IAC group than in the pre-invasive group (*P’* < 0.025).

## 4 Discussion

In this prospective single-center cohort of patients who underwent surgical resection for SPNs, we evaluated the complementary value of 1024-matrix UHRCT, urinary CEM, and serum 7-TAAbs for preoperative nodule characterization. Our findings indicate that, although UHRCT alone exhibited excellent diagnostic capability (AUC = 0.831), multimodal integration provided additional diagnostic information, particularly when implemented using a probability-based logistic regression framework. Notably, despite the modest sensitivity of the individual liquid biopsy markers for early lung cancer detection, they demonstrated a complementary role in providing biological signals associated with invasive adenocarcinoma pathology. These findings support the clinical value of an “imaging–immune–metabolic” combined diagnostic approach, suggesting that it may facilitate more accurate preoperative risk-stratification support than morphological assessment alone, particularly when differentiating high-risk subsolid nodules.

Importantly, the combined strategies in the original analysis were based on a parallel-testing rule, in which a combined result was considered positive if any constituent test yielded a positive result. This sensitivity-oriented rule increased sensitivity but markedly reduced specificity, indicating a potential risk of false-positive classifications. Therefore, we further constructed probability-based logistic regression models and evaluated their threshold-dependent diagnostic performance. This approach allowed a more balanced interpretation of multimodal testing than a simple “any-positive” rule.

When comparing modalities, UHRCT demonstrated superior diagnostic performance (AUC = 0.831), whereas the standalone performance of the liquid biopsy markers was limited. In the present study, the AUCs for the urinary metabolic index and 7-TAAbs were only 0.605 and 0.558, respectively. This limitation is consistent with the pathophysiology of early lung adenocarcinoma: the tumor burden in lesions presenting as GGNs is typically low and may be insufficient to release detectable levels of biomarkers into the circulation using conventional assays (15). In addition, early-stage adenocarcinoma lesions may show heterogeneous metabolic activity and variable humoral immune responses, which may further limit the sensitivity of urine- or serum-based biomarkers when used alone (10–13). Therefore, these biomarkers should not be interpreted as replacements for imaging, but rather as complementary biological indicators.

The interpretation of multimodal integration depends on how the combined result is defined. Under the parallel-rule strategy, multimodal testing maximized sensitivity but substantially reduced specificity. In contrast, the probability-based integrated model generated continuous predicted probabilities, allowing threshold-dependent adjustment of sensitivity and specificity. In the present analysis, the fully integrated model achieved the highest AUC, although the incremental improvement over the clinical-UHRCT model was modest. These findings suggest that UHRCT and clinical variables contributed the majority of the diagnostic information, whereas urinary CEM and 7-TAAbs provided relatively limited additional diagnostic value (16–18).

In the paired imaging comparison, UHRCT demonstrated significant advantages over conventional HRCT. Our analysis revealed that compared with conventional HRCT, UHRCT improved sensitivity from 68.0% to 92.0% and specificity from 73.3% to 90.0%. This improvement likely stems from its enhanced spatial resolution. Reconstruction using a 1024×1024 matrix reduces pixel size, enabling clear visualization of fine anatomical structures (19, 20). This capability has important clinical implications because accurate preoperative assessment of lung adenocarcinoma–lineage lesions depends on the identification of invasive features within GGNs, such as the extent of micro-solid components, marginal spiculation, and vascular convergence signs (21–23). UHRCT depicts these features more reliably than conventional CT, where they may be obscured, thereby improving diagnostic performance. Consequently, UHRCT may improve preoperative risk stratification and provide a more reliable basis for selecting the appropriate surgical approach (e.g., sublobar resection vs. lobectomy).

Beyond diagnostic efficacy, this study revealed the potential value of liquid biopsy modalities in assessing biological features associated with tumor invasiveness. Subgroup analysis demonstrated that the positive rates of the urinary metabolic index and 7-TAAbs in patients with IAC (57.6% and 53.8%, respectively) were significantly higher than those in the pre-invasive (AIS/MIA) group (~31%, *P* < 0.005). This finding suggests that elevated fluid biomarkers may reflect transition to a more aggressive stage. In clinical practice, determining the degree of GGN invasion solely by imaging remains difficult, especially when distinguishing MIA from early IAC (24). In this context, liquid biopsy may serve as a powerful adjunct. For nodules showing pure ground-glass or micro-invasive features on imaging but positive fluid markers, surgeons should remain alert to the risk of underestimated potential invasiveness. Such preoperative information may guide targeted intraoperative frozen-section sampling and support more cautious decisions regarding the extent of resection when frozen-section results are equivocal, thereby reducing the risk of inadequate margins due to underestimation of lesion invasiveness. However, because this analysis was based on subgroup comparisons of biomarker positivity rather than on a dedicated invasiveness prediction model, these findings should be interpreted as evidence of associations with invasive pathology rather than definitive evidence of independent preoperative prediction.

This study has some limitations. First, as a single-center study, the sample size was relatively small, and the participants were patients scheduled for surgery. This selection bias resulted in a higher proportion of malignant nodules than would be expected in a general screening population; therefore, the PPV reported in this study may be overestimated, and caution is warranted when generalizing conclusions to general outpatient screening populations. Specifically, the cohort included 164 malignant nodules and only 19 benign lesions, resulting in a malignancy prevalence of 89.6%. Such a high prevalence may reduce the stability of specificity and NPV estimates and limits the external validity of the model in screening or routine outpatient SPN populations. Second, although an association was observed between the urinary metabolic index and tumor invasiveness, the underlying molecular biological mechanisms require further investigation through experimental studies. Third, although probability-based logistic regression models and exploratory decision-curve analysis were incorporated, these analyses were not externally validated and should therefore be considered exploratory. Finally, long-term follow-up data were unavailable. Future multi-center, large-scale longitudinal studies are needed to determine whether this multimodal model improves long-term outcomes, including overall survival.

## 5 Conclusion

In summary, this prospective single-center study suggests that 1024-matrix UHRCT, combined with urinary CEM and serum 7-TAAbs, may provide a complementary multimodal approach for the preoperative risk stratification of surgically treated SPNs. UHRCT reduces missed diagnoses through improved spatial resolution, while liquid biopsy provides complementary information by reflecting tumor biological features associated with invasive adenocarcinoma pathology. The probability-based integrated model may offer a more balanced diagnostic framework than a simple parallel-rule strategy. However, given the high prevalence of malignancy, the limited number of benign lesions, and the lack of external validation, these findings should be considered exploratory and require confirmation in broader outpatient and screening cohorts.

## Data Availability

The raw data supporting the conclusions of this article will be made available by the authors, without undue reservation.
