# Supplementary material for: Preoperative evaluation of solitary pulmonary nodules and adenocarcinoma invasiveness using ultra-high-resolution computed tomography and multidimensional liquid biopsy: a prospective exploratory study
Source: Front Oncol. 2026 Jul 15;16:1818928. doi: 10.3389/fonc.2026.1818928 (PMC13414198; doi:10.3389/fonc.2026.1818928)
Supplement: Supplementary file 1 [file Table1.docx]

Supplementary Material

## Supplementary Figures

| **Predictor** | **OR** | **95% CI** | **P value** |
| --- | --- | --- | --- |
| Age | 1.05 | 0.93–1.18 | 0.413 |
| Male sex | 4.81 | 0.77–30.09 | 0.093 |
| Smoking history | 4.29 | 0.70–26.34 | 0.116 |
| Nodule size | 4.83 | 1.31–17.75 | 0.018 |
| UHRCT positive | 66.04 | 9.11–478.62 | <0.001 |
| Urine CEM positive | 8.25 | 1.33–51.33 | 0.024 |
| 7-TAAbs positive | 4.97 | 1.05–23.43 | 0.043 |

**Supplementary Table S1.** Multivariable logistic regression analysis of predictors for malignant SPNs
